# MP-V1 from the Venom of Social Wasp *Vespula vulgaris* Is a *de Novo* Type of Mastoparan that Displays Superior Antimicrobial Activities

**DOI:** 10.3390/molecules21040512

**Published:** 2016-04-19

**Authors:** Yangseon Kim, Minky Son, Eun-Young Noh, Soonok Kim, Changmu Kim, Joo-Hong Yeo, Chanin Park, Keun Woo Lee, Woo Young Bang

**Affiliations:** 1 National Institute of Biological Resources (NIBR), Environmental Research Complex, Incheon 404-708, Korea; nimitzdr93@gmail.com (Y.K.); rey0426@korea.kr (E.-Y.N.); sokim90@korea.kr (S.K.); snubull@korea.kr (C.K.); y1208@korea.kr (J.-H.Y.); 2 Division of Applied Life Science (BK21 Plus) & Plant Molecular Biology and Biotechnology Research Center (PMBBRC), Gyeongsang National University (GNU), 501 Jinju-daero, Jinju 660-701, Korea; minky@gnu.ac.kr (M.S.); chaninpark0806@gmail.com (C.P.); kwlee@gnu.ac.kr (K.W.L.)

**Keywords:** antimicrobial activity, mastoparan, MP-V1, peptide, venom, wasp

## Abstract

Mastoparans from the venom of social wasps have attracted considerable attention as effective antibiotic candidates. In this study, mastoparan V1 (MP-V1) from *Vespula vulgaris* was first disclosed to have a peptide amino acid sequence distinct from typical mastoparans and its biochemical properties and antimicrobial effects were compared with those of typical mastoparans MP-L, -X(V) and -B. Circular dichroism (CD) spectroscopy revealed that MP-V1 and -X(V) form more stable α-helical conformations in lipid membrane-like environments than MP-L and -B. In parallel, these two also showed more effective antimicrobial activities against the pathogens than did MP-L and -B. Although MP-V1 had a less stable α-helical conformation than MP-X(V), it showed stronger antimicrobial effects against *Streptococcus mutans* and *Salmonella enterica* than MP-X(V). In the meantime, analysis of hemolytic activity revealed a range of doses (~50 μM) that exhibited little potent cytotoxicity on human erythrocytes. Finally, the atypical MP-V1 peptide amino acid sequence provided important clues to understanding its antimicrobial mechanism from a structural perspective. Therefore, it has been concluded that MP-V1 is a *de novo* type of mastoparan with superior antimicrobial activities against both pathogenic bacteria and fungi, which may be useful in developing multipurpose antimicrobial drugs against infectious diseases.

## 1. Introduction

Recent studies have highlighted the discovery and modification of antimicrobial peptides (AMPs) for use as therapeutic tools against infectious diseases [1,2,3]. The AMPs also have exhibited potential as antibiotic candidates that can be used to overcome the microbial resistance challenge [3,4,5]. The AMPs are small peptides ranging from 10 to 40 amino acids in size and have common features, such as cationicity and amphipathicity [1,5]. In addition, based on their structures the AMPs are further classified into four groups, such as α-helical peptides, β-sheet peptides, extended peptides and loop peptides [1,5]. Although AMPs’ exact mechanism of action is not fully understood, they are known to cause microbial membrane damage via either pore formation through a barrel-stave or a toroidal pore mechanism or through a non-pore carpet-like mechanism [5].

**Figure 1:**
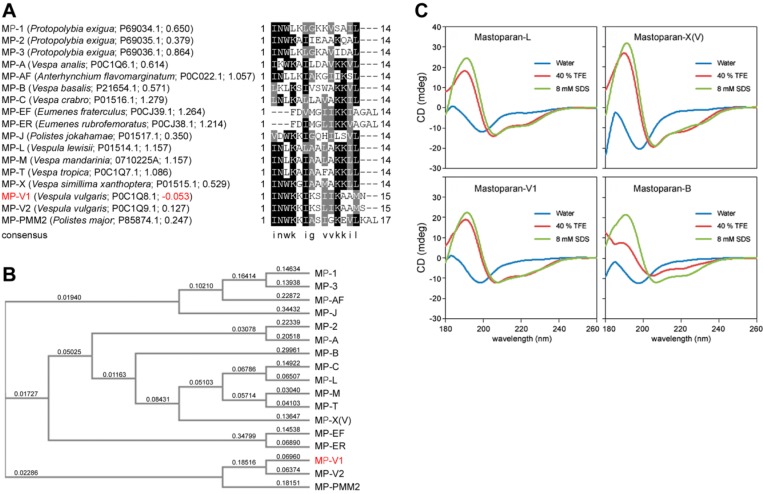
Comparison of the primary and secondary structures of MP-V1 with those of other mastoparans. (**A**) Amino acid sequence alignment for mastoparans found in the NCBI protein database. The parentheses include a wasp scientific name, an accession number and a GRAVY value. Identical or similar residues are shown in black or gray shaded boxes, respectively. The all mastoparans are identified to be C-terminally amidated; (**B**) Phylogenetic tree of the mastoparans shown in (**A**). Because MP-X was identified from *Vespa velutina flavitarsus* as well as *Vespa simillima xanthoptera*, it was represented as MP-X(V) afterward; (**C**) Circular dichroism (CD) spectra of the mastoparans synthesized, MP-V1, MP-L, MP-X(V) and MP-B. The synthetic mastoparans contain an acidic C terminus without amidation.

Mastoparans derived from the venom of social wasps are representative AMPs and originally have been known to have mast cell-degranulating activity, playing a defensive role by causing allergic reactions in envenomed predators [6]. However, there is growing scientific evidence that the mastoparans are beneficial to the antimicrobial and antiviral effects on pathogens [7,8,9,10] and the anti-cancer effects [11]. The mastoparans are commonly cationic tetradecapeptides, having C-terminal amide capping, and form amphipathic α-helices in lipid membranes [12,13]. Notably, it was observed in this study that the mastoparan V1 (MP-V1) from the venom of *Vespula vulgaris* [14] has an unusual peptide amino acid sequence compared to typical mastoparans; it consists of 15 amino acids, including an additional 15th asparagine, and possesses a lysine with a charged side-chain at the 7th position in the middle of the peptide (Figure 1). This sequence is different from that of typical mastoparans [15,16,17], which are composed of 14 amino acids and have hydrophobic residues in the middle [12,13]. This unusual sequence may form a unique conformation, different from those of other mastoparans, which may affect the antimicrobial activity compared to typical mastoparans. Therefore, MP-V1 has interesting potential for the discovery or design of new antimicrobial drugs against various pathogens, including multidrug-resistant microbes, but its biochemical properties and antimicrobial roles in pathogens remain uncharacterized. To the best of our knowledge, this report is the first that addresses these issues.

In this study, we first characterized the biochemical properties and antimicrobial activities of MP-V1 by conducting a comparison of the four peptides, MP-V1, -L, -B and -X(V) and, consequently, identified that MP-V1 is a *de novo* type of mastoparan displaying superior antimicrobial activities. Therefore, our study supplies important information regarding the discovery and design of new antimicrobial peptides with improved performance.

## 2. Results

### 2.1. Identification of MP-V1 as a de Novo Type of Mastoparan

Generally, typical mastoparans are composed of 14 residues (Figure 1A), but we found the three unusual mastoparans with more than 15 residues, such as MP-V1 [14], MP-V2, a paralog of MP-V1 [14], and MP-PMM2 [18], from the NCBI protein database [19] (Figure 1A,B). Noticeably, MP-V1 derived from the venom of *Vespula vulgaris* was found to be an atypical mastoparan that shows the lowest hydrophobicity with the grand average of hydropathicity (GRAVY) value, −0.053, among all mastoparans shown in Figure 1A, that contains an additional asparagine, a polar amino acid, as the 15th residue, and that has a 7th lysine with a charged side-chain in the middle of the peptide, where the typical mastoparans include hydrophobic amino acids (Figure 1A). Therefore, it is suggested that MP-V1 may have distinct biochemical and antimicrobial features from the typical mastoparans, which is further supported by subsequent experiments with the mastoparans, MP-V1, MP-L, MP-X(V) and MP-B, synthesized as described in the “Materials and Methods” section.

### 2.2. Comparison of the Secondary Structure of MP-V1 with Those of Other Mastoparans

To investigate the secondary structures of mastoparans, circular dichroism (CD) spectroscopy was carried out in different environments. The CD spectra of all the synthetic mastoparans in water showed random-coil character (Figure 1C). However, in the presence of 8 mM SDS and 40% TFE, the CD spectra of all the mastoparans exhibited the α-helical character (Figure 1C). Percent α-helix calculated for the mastoparans in different environments revealed that mastoparans tend to form an α-helix in 8 mM SDS or 40% TFE, whereas they tend to form a random-coil conformation in water (Table 1). Notably, among all mastoparans, MP-X(V) showed the highest percent α-helix in 8 mM SDS or 40% TFE, with MP-V1 also exhibiting a somewhat high percent of α-helix compared with MP-L and MP-B (Table 1).

### 2.3. Comparison of Hemolytic Activity of MP-V1 with the One of Other Mastoparans

**Figure 2:**
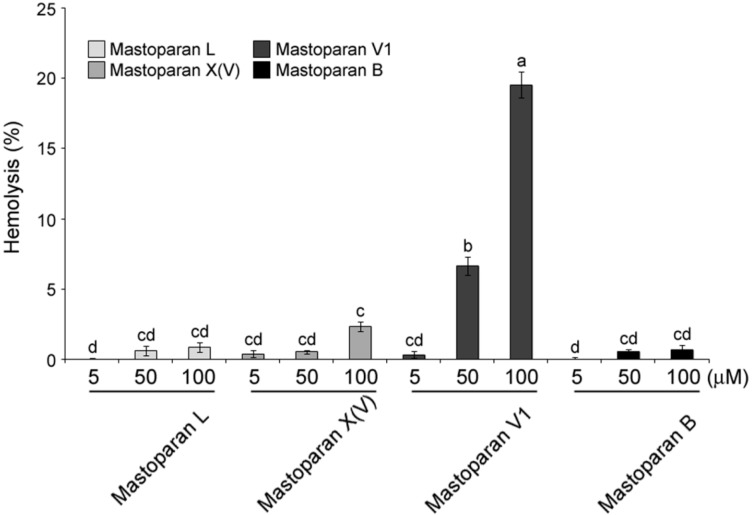
Hemolytic activity of mastoparans. The hemolytic activity was analyzed in human erythrocytes. The absorbance measured at 540 nm from the supernatants of lysed RBC in PBS or 0.1% Triton X-100 were determined as 0% and 100%, respectively. Data are means ± S.E. (*n* = 3). Different letters (a, b, c and d) indicate significant differences by the ANOVA/Tukey-HSD (*p* < 0.05).

Hemolytic activity of the synthetic mastoparans was examined with 5 to 100 μM doses, as shown in Figure 2. Generally, there was notably little hemolysis in human erythrocytes treated with MP-L, -X(V) and -B. However, when the erythrocytes were treated with MP-V1, dose-dependent hemolysis was observed. The MP-V1 showed a weak hemolytic activity of 6.6% at 50 μM, whereas it exhibited a more potent hemolytic activity of approximately 20% on the erythrocytes as its concentration was increased to 100 μM (Figure 2). This result indicates that except for the 100 μM MP-V1, mastoparans do not exhibit potent cytotoxicity on human erythrocytes.

### 2.4. Comparison of Antimicrobial Activities of MP-V1 with Other Mastoparans

**Figure 3:**
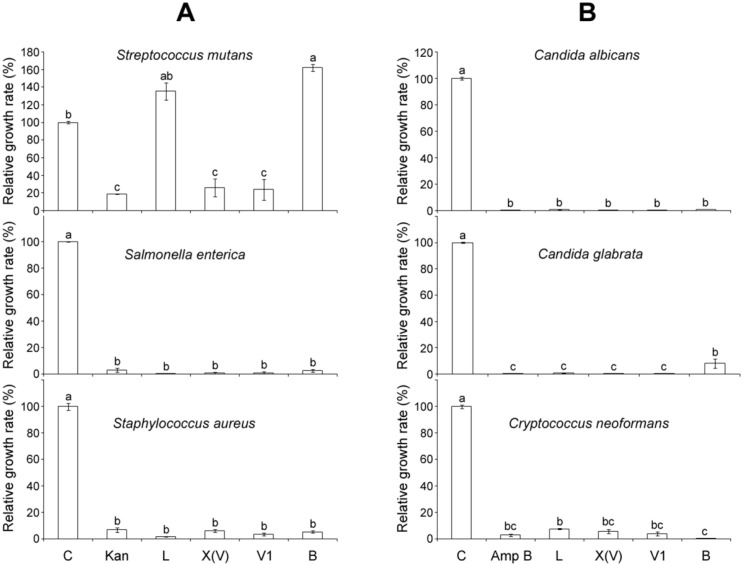
Effects of 100 μM mastoparans on pathogen growth. (**A**) The bacteria, *Streptococcus mutans*, *Salmonella enterica* and *Staphylococcus aureus*; and (**B**) the fungi, *Candida albicans*, *Candida glabrata* and *Cryptococcus neoformans* were treated with 100 μM MP-L, -X(V), -V1 or -B to compare their effects on pathogen growth. The optical density (OD) at 600 nm was taken as a measure of pathogen growth. The 50 μg/mL Kanamycin (Kan) and 10 μg/mL Amphotericin B (Amp B) were used as positive controls for bacteria and fungi, respectively. Data are means ± S.E. (*n* = 3). Different letters (a, b and c) indicate significant differences by the ANOVA/Tukey-HSD (*p* < 0.05).

Antimicrobial activity of mastoparans was examined in the pathogenic bacteria *S. mutans*, *S. enterica* and *S. aureus* and in the pathogenic fungi *C. albicans*, *C. glabrata* and *C. neoformans* with a 100 μM dose, as shown in Figure 3. All mastoparans completely inhibited the growth of most of pathogens at the 100 μM dose. However, MP-L and MP-B showed no antimicrobial activity in *S. mutans*, whereas MP-X(V) and MP-V1 exhibited significantly inhibitory effects on the growth of *S. mutans*, almost on the level of kanamycin, the positive control (Figure 3A).

**Figure 4:**
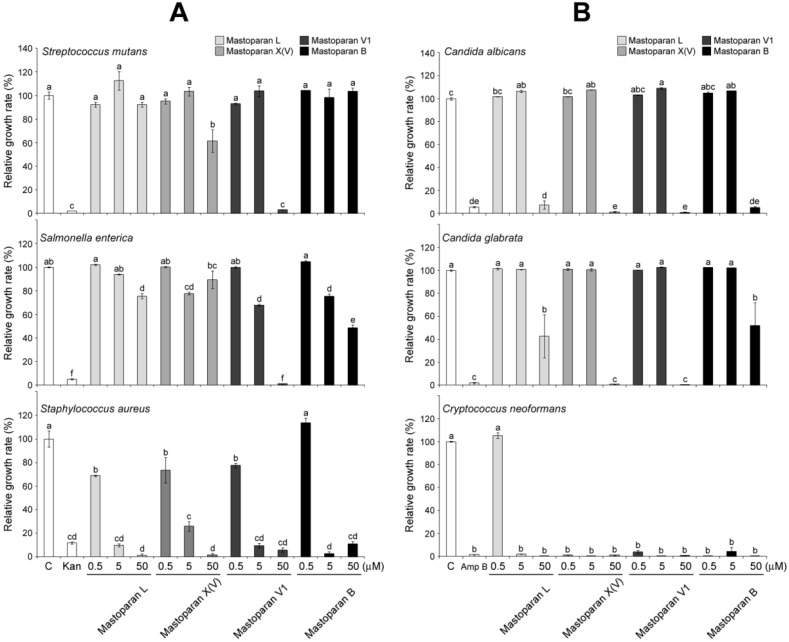
Comparative effects of various doses of mastoparans on pathogen growth. (**A**) The bacteria, *Streptococcus mutans*, *Salmonella enterica* and *Staphylococcus aureus*; and (**B**) the fungi, *Candida albicans*, *Candida glabrata* and *Cryptococcus neoformans*, were treated with various doses (0.5 μM, 5 μM and 50 μM) of MP-L, -X(V), -V1 or -B to compare their effects on pathogen growth. The optical density (OD) at 600 nm was taken as a measure of pathogen growth. The 50 μg/mL Kanamycin (Kan) and 10 μg/mL Amphotericin B (Amp B) were used as positive controls for bacteria and fungi, respectively. Data are means ± S.E. (*n* = 3). Different letters (a, b, c, d and e) indicate significant differences by the ANOVA/Tukey-HSD (*p* < 0.05).

Furthermore, anti-bacterial activities were compared among all of the mastoparans using various doses, such as 0.5 μM, 5 μM and 50 μM, as shown in Figure 4A. In *S. mutans*, only 50 μM MP-V1 completely inhibited the bacterial growth, while 50 μM MP-X(V) showed a partially inhibitory effect on the growth and the MP-L and MP-B exhibited no effects (the upper graph in Figure 4A). In *S. enterica*, MP-V1 exhibited dose-dependent anti-bacterial activity and completely inhibited growth at 50 μM, while the others showed partial activity (the middle graph in Figure 4A). In *S. aureus*, all mastoparans showed dose-dependent anti-bacterial activities and exhibited almost complete growth inhibition at 50 μM (the lower graph in Figure 4A).

Finally, anti-fungal activities were compared among all mastoparans using various doses, as shown in Figure 4B. In *C. albicans*, all mastoparans completely inhibited fungal growth at 50 μM (the upper graph in Figure 4B). In *C. glabrata*, MP-V1 and MP-X(V) showed complete growth inhibition at 50 μM, whereas MP-L and MP-B showed significant partial inhibition at 50 μM (the middle graph in Figure 4B). In *C. neoformans*, except for the 0.5 μM MP-L, all mastoparans completely inhibited fungal growth at all doses (the lower graph in Figure 4B). Taken together, these results indicate that 50 μM MP-V1 can completely inhibit the growth of all pathogens used in this study, suggesting that it is a promising candidate for the discovery of new anti-pathogenic drugs with high efficiency.

## 3. Discussion

### 3.1. The Mechanism of Action of Typical Mastoparans on Microbial Membranes

Mastoparans share a set of common biophysical features for their antimicrobial activity; they have a net positive charge, mediating their electrostatic attraction to the negatively charged microbial surfaces, and form an amphipathic α-helix that results in a face displaying the hydrophobic residues for embedding into the hydrophobic part of the microbial membrane interface [13,20]. During their interaction with the membrane, the mastoparans have been known to cause membrane disruption through a mechanism such as the carpet model, the toroidal model or the barrel-stave model [5]. Recently, a mass spectrometry study strongly supported the hypothesis that mastoparans cause membrane perturbation through the carpet model [21]. In this model, a mastoparan interacts in parallel to the membrane surface without complete internalization into the lipid layer and both its N- and C-termini remain outside of the membrane [21]. Especially during its interaction with the membrane, the mastoparan forms an α-helix to optimize amphipathicity, changing conformations in the middle of the peptide in order to make its conformation more energetically favorable and leading to bilayer destabilization, while under water conditions, it forms a randomly coiled chain [21]. Noticeably, the C-terminal amide capping of the mastoparan promotes the stabilization of its helical conformations, allowing an intense embedment with animal and bacterial cell membranes [21]. Therefore, net positive charge, hydrophobicity and stabilization of the helical conformation should be considered to improve the antimicrobial activity of mastoparans.

### 3.2. Unusual MP-V1 Peptide Amino Acid Sequence Responsible for High-Efficiency Antimicrobial Activity

MP-V1 showed superior antimicrobial activities compared with other mastoparans studied (Figure 3 and Figure 4). Considering its low hydrophobicity in the GRAVY calculation (Figure 1A), how does the MP-V1 exhibit the astonishing antimicrobial activities compared with other mastoparans? The MP-V1 was shown to contain an additional asparagine at the 15th residue, distinctly different from typical mastoparans composed of 14 amino acids. It is generally known that asparagine can play the role of end capping in stabilization of the helical conformation because its side-chain amide group can form a hydrogen bond interaction with a peptide backbone [22,23]. Accordingly, the side-chain amide group of the 15th asparagine may function in the C-terminal capping, promoting the α-helical stabilization of MP-V1. This is supported by the CD spectra comparison of synthetic mastoparans containing an acidic C terminus without amidation; MP-V1 formed a more stable helical conformation in the membrane-like environments, such as 8 mM SDS and 40% TFE (Figure 1C and Table 1), with higher antimicrobial activities (Figure 3 and Figure 4) than MP-L and MP-B. Moreover, MP-V1 showed more potent hemolytic activity (Figure 2) as well as antimicrobial activity, indicating a decrease in microbial membrane selectivity. A previous study reported that the C-terminal amidation causes the loss of membrane selectivity, leading to more perturbations of both animal and microbial membranes than its acidic congener, despite promoting the stabilization of the peptide [21]. Thus, this also supports that the 15th asparagine in MP-V1 enhances the stabilization of the helical conformation but leads to a decrease in membrane selectivity.

Although MP-V1 forms a less stable helical conformation in membrane environments than MP-X(V) (Figure 1C and Table 1), it exhibits higher antimicrobial activity on *S. mutans* and *S. enterica* than MP-X(V) (Figure 4A). This may be caused by the 7th lysine in the MP-V1 peptide (Figure 1A and Figure S3). Generally, typical mastoparans have hydrophobic residues in the middle of peptide, embedded into the hydrophobic part of the microbial membrane interface, leading to membrane perturbation [12,13]. The insertion of charged side-chains of residues, such as aspartic acid or lysine, into the hydrophobic part of the membrane causes the occurrence of frequent conformational changes in the middle region of the peptide, resulting in membrane destabilization [21]. Accordingly, the unusual presence of the 7th lysine in MP-V1 may cause more frequent conformational changes than MP-X(V), resulting in more intense membrane perturbation.

### 3.3. Identification of C-Terminal Acidic Mastoparans as Multipurpose Antibiotics

The pathogens used in this study cause the following infectious diseases; the pathogenic bacteria *S. mutans*, *S. enterica* and *S. aureus* cause tooth decay, food poisoning and pneumonia, respectively, and the pathogenic fungi *C. albicans* and *C. glabrata*, and *C. neoformans* cause candidiasis and cryptococcosis, respectively. The mastoparans, synthesized without C-terminal amidation, significantly inhibited the growth of all pathogens listed above (Figure 3 and Figure 4) with little hemolysis on human erythrocytes (Figure 2), indicating that their acidic C-terminus may enhance the bacterial membrane selectivity. For example, MP-V1 and MP-X(V) completely inhibited the growth of all pathogens above at 50 μM and 100 μM doses, respectively (Figure 3 and Figure 4), showing little cytotoxicity on human erythrocytes (Figure 2). MP-L and MP-B also completely inhibited the growth of all the pathogens, except for *S. mutans*, at a 100 μM dose (Figure 3 and Figure 4), showing no cytotoxicity on human erythrocytes (Figure 2). Only the 100 μM MP-V1 showed potent hemolytic activity (Figure 2). Therefore, the mastoparans examined in this study can be utilized as multipurpose antibiotics for the prevention or treatment of various diseases described above with no cytotoxicity on the human body. 

In conclusion, we characterized the biochemical properties and antimicrobial activities of the MP-V1 peptide derived from the venom of *Vespula vulgaris* through comparison with those of other typical mastoparans. Consequently, MP-V1 was identified to be a *de novo* type of mastoparan displaying superior antimicrobial activities. In addition, MP-L, -X(V) and -B showed efficient antimicrobial activities with no cytotoxicity on human erythrocytes. Altogether, our results provide important insights into the exploration and design of new antimicrobial peptides for the development of highly efficient antibiotics. 

## 4. Materials and Methods 

### 4.1. Biological Materials

The three pathogenic fungi used in this study, *Candida albicans*, *Candida glabrata* and *Cryptococcus neoformans*, were obtained from Yongsun Bahn (Yonsei University, Seoul, Korea). The three pathogenic bacteria used, *Salmonella enterica* (ATCC 39183), *Staphylococcus aureus* (KCTC 1621) and *Streptococcus mutans* (KCTC 3065), were purchased from the Korean Collection for Type Cultures (KCTC, Daejeon, Korea) and the American Type Culture Collection (ATCC, Manassas, VA, USA). Human red blood cells (RBCs) were kindly supplied by Young Ho Koh (Hallym University, Anyang, Korea) [24].

### 4.2. Bioinformatic Analyses

The amino acid sequences of mastoparans in Figure 1A were identified from the NCBI protein database [19] by using mastoparan as a keyword. Subsequently, the amino acid sequences were used for the alignment of amino acid sequences and the preparation of phylogenetic tree, done by the Multiple Sequence Alignment and the Neighbour-joining plot tools of the Clustal Omega program (EMBL-EBI, Cambridgeshire, UK), respectively. The grand average of hydropathicity (GRAVY) calculation was performed by the ProtParam [25].

### 4.3. Peptide Synthesis and Purification

All peptides were prepared by Fmoc SPPS methods using Wang resin (100 mg) with an initial loading of 0.61 mmol/g unless otherwise noted. Resins were washed in DMF for 45 min prior to synthesis to ensure proper swelling. For sequence extension, the Fmoc-protected amino acid (5 eq.) was activated by treatment with HBTU (5.0 eq.), HOBt (5.0 eq.) and DIEA (10 eq.) in DMF (2 mL, 0.15 mM) for 2 min. This solution was added to the free amine on the resin, and the coupling reaction was allowed to proceed for 1 h with Vortex stirring. After washing with DMF, Fmoc deprotection was achieved with 20% piperidine in DMF (1 × 10 min, 2 × 3 min). The resin was washed with DMF (3 × 3 min), and the process was repeated for the next amino acid. Linear peptides were cleaved from the resin with 5% triisopropylsilane (TIS) and 5% H_2_O in trifluoroacetic acid (TFA, approximately 2 mL of TFA per 100 mg of resin) for 2 h. The cleavage cocktail was mixed with cold ether to precipitate the peptide and later centrifuged. Preparative reverse-phase HPLC analysis (RP-HPLC) was carried out on the Vydac C_18_ column (15 μm, 20 mm × 250 mm) using a 0% to 90% water/acetonitrile gradient in the presence of 0.05% TFA. The final purity of the peptides (>95%) was assessed by RP-HPLC on an analytical Vydac C_18_ column (4.6 mm × 250 mm, 300 Å, 5 μm particle size), as shown in Figure S1. The molecular masses of purified peptides were determined using matrix-assisted laser-desorption ionization-time-of-flight mass spectrometry (MALDI-TOF MS) (KBSI, Ochang, Korea), as shown in Figure S2.

### 4.4. Circular Dichroism Spectroscopy

Circular dichroism spectra were measured on a J-715 spectropolarimeter (JASCO International Co. Ltd., Tokyo, Japan) at 298K using a quartz cuvette of 0.1 cm path length. The CD spectra at 0.1 mg/mL peptide concentration was recorded in three different environments: water, 40% 2,2,2-trifluoroethanol solution (TFE) and 8 mM sodium dodecylsulfate (SDS). The spectra were recorded from 260 nm to 180 nm in triplicate. Data were recorded at a scan speed of 200 nm/min, bandwidth of 1.0 nm, 1 s response and 0.1 nm resolution. The percent α-helix was calculated using the following equation: % α-helix = −100 × (θ_222nm_ + 3000)/33,000, as described previously [26].

### 4.5. Pathogen Growth and Antimicrobial Test

The fungi *C. albicans*, *C. glabrata* and *C. neoformans* were cultured on Sabouraud dextrose agar (SDA) plates at 30 °C for 48 h. The bacteria *S. enterica*, *S. aureus*, *S. mutans* were cultured at 37 °C for 24 h on a trypticase soy agar plate, nutrient agar plate, and brain heart infusion agar plate, respectively. 

The antimicrobial activity was tested by standard micro-assays using conventional sterile polystyrene microplates [27]. Each well of the microplate was filled with 100 µL of sterile media and approximately 50 µL of inoculum and 50 µL of peptide solution were added to each well at final concentrations ranging from 0.5 to 100 µM. The final inoculum concentrations were approximately 1.0 × 10^6^ conidia/mL for *C. albicans*, *C. glabrata* and C*. neoformans*, and 2.5 × 10^6^ CFU/mL for *S. aureus*, *S. enterica* and *S. mutans.* Media containing only 50 µL of inoculums and 50 µL of 1% dimethyl sulfoxide without the peptide solution was used as the control treatment and media containing only 50 µL of 1% dimethyl sulfoxide without the inoculum was used as a solvent control. Amphotericin B and kanamycin were added to the microorganisms as positive controls for growth inhibition. The microplates were incubated for 24 h at 37 °C and 30 °C for *S. aureus*, *S. enterica* and *S. mutans* and were incubated for 48 h at 30 °C for *C. albicans*, *C. glabrata* and C*. neoformans*. The microbial growth was determined by reading the OD at 600 nm using a multiplate reader. All experiments were repeated three times. The percentage of microbial growth in response to different peptide solutions was calculated from the control treatment using the following equation: Relative growth rate (%) = ((Control_Abs_ − Treatment_Abs_)/Control_Abs_) × 100%, where Control_Abs_ is the absorbance of the control and Treatment_Abs_ is the absorbance of the specific peptide solution.

### 4.6. Hemolytic Activity

The hemolytic activity assay was performed as previously described [26] with slight modification. Human red blood cells (RBCs) were used to assess the hemolytic activities of the synthesized venom peptides from the four social wasp species. The RBCs were washed three times for 5 min at 960 rpm with phosphate-buffered saline (PBS) and resuspended to 10% in the same buffer. One hundred microliters of various concentrations (10 to 200 μM) of synthesized peptides were incubated with 100 μL of RBCs for 30 min at 37 °C. After centrifugation for 5 min at 960 rpm, the OD values of the supernatants were measured at 540 nm using a microplate reader (Synergy HT, Bio-Tek Instruments, Inc., Winooski, VT, USA). The relative hemolytic activity was determined by establishing the activity of PBS as 0% and that of 0.1% Triton X-100 as 100%. The hemolysis (%) was calculated using the following equation: Hemolysis (%) = [(A_MP_ − A_PBS_)/(A_0.1% Triton X-100_ − A_PBS_)] × 100%. Results are expressed as means ± SE of three replicates.

### 4.7. Statistical Analysis

The one-way analysis of variance (ANOVA) was followed by Tukey-Kramer HSD using InfoStat version 2012 software (National University of Cordoba, Cordoba, Argentina). Results are expressed as means ± standard errors (S.E.) of at least three independent experiments. Different letters indicate significant differences (*p* < 0.05).

## Figures and Tables

**Table 1:** Percent α-helix of mastoparans in different environments.

Buffers	MP-L		MP-X(V)		MP-V1		MP-B	
	[θ]222	% α-Helix	[θ]222	% α-Helix	[θ]222	% α-Helix	[θ]222	% α-Helix
Water	−2792.88	r.c.	−1828.33	r.c.	−1480.77	r.c.	−1477.65	r.c.
8 mM SDS	−9450.15	19.55	−11459.40	25.63	−9965.03	21.11	−9963.52	21.10
40% TFE	−8896.83	17.87	−12974.80	30.23	−10261.60	22.00	−5615.77	7.93

The r.c. indicates random coil conformation.

## Supplementary Materials

The following are available online at www.mdpi.com/1420-3049/21/4/512/s1, Figure S1: Reverse-phase HPLC (RP-HPLC) analysis of the four mastoparans, Figure S2: Matrix-assisted laser-desorption ionization-time-of-flight mass (MALDI-TOF MS) analysis of the four mastoparans, Figure S3: Structural models of mastoparans.

## Abbreviations

The following abbreviations are used in this manuscript:

AMP	antimicrobial peptide
CD	circular dichroism
MP	mastoparan
TFE	2,2,2-trifluoroethanol
