# Supplementary material for: MP-V1 from the Venom of Social Wasp Vespula vulgaris Is a de Novo Type of Mastoparan that Displays Superior Antimicrobial Activities
Source: Molecules. 2016 Apr 19;21(4):512. doi: 10.3390/molecules21040512 (PMC6273832; doi:10.3390/molecules21040512)
Supplement: Supplementary file 1 [file molecules-21-00512-s001.pdf]

Supplementary Materials: MP-V1 from the Venom of Social Wasp *Vespula vulgaris* is a *de Novo* Type of Mastoparan that Displays Superior Antimicrobial Activities

Yangseon Kim, Minky Son, Eun-Young Noh, Soonok Kim, Changmu Kim, Joo-Hong Yeo, Chanin Park, Keun Woo Lee and Woo Young Bang

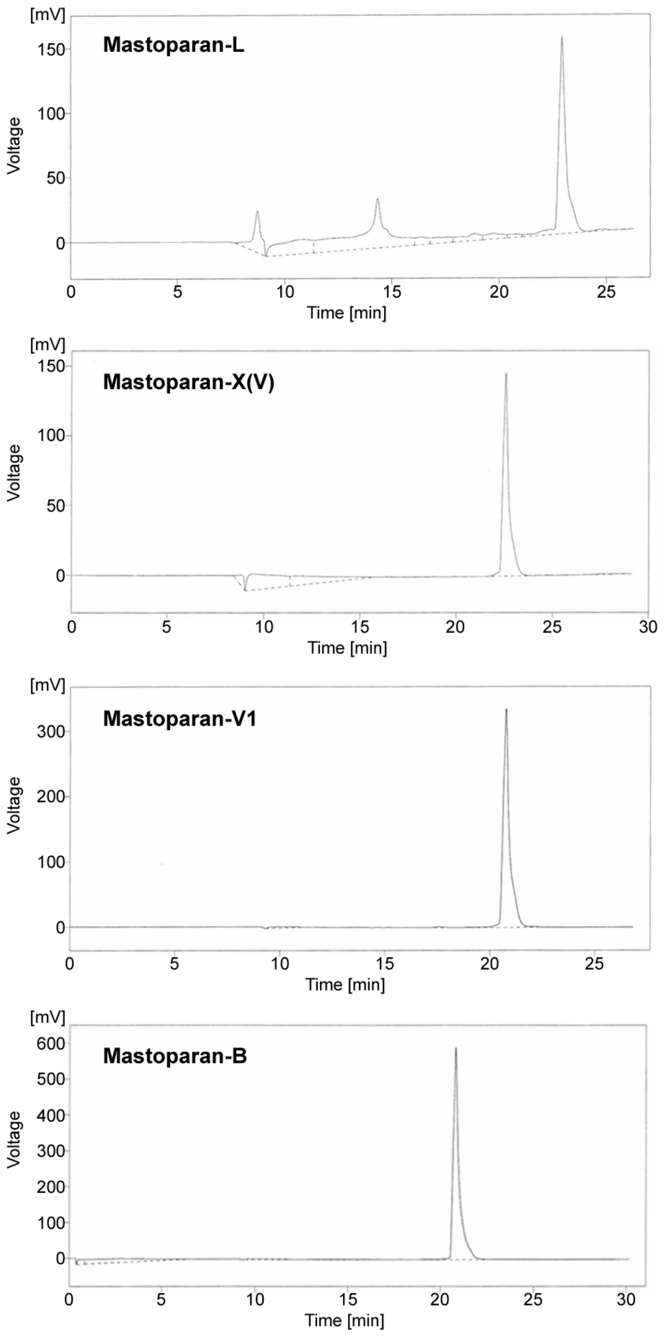

Figure S1. Reverse-phase HPLC (RP-HPLC) analysis of the four mastoparans. The RP-HPLC was carried out on the Vydac C_{18} column (15 µm, 20 mm × 250 mm) using 0%–90% water/acetonitrile gradient in the presence of 0.05% TFA. The final purity of the peptides (>95%) was assessed by RP-HPLC on an analytical Vydac C_{18} column (4.6 mm × 250 mm, 300 Å, 5 µm particle size).

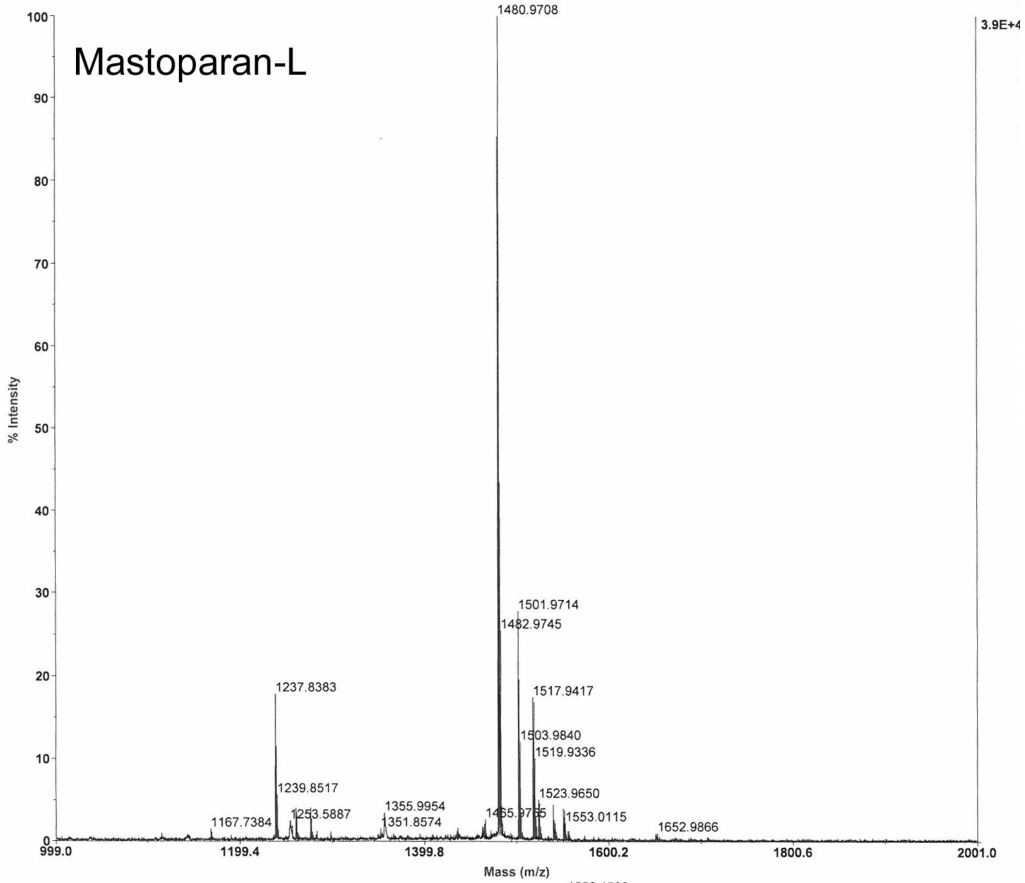
Mastoparan-L
1480.9708
3.9E+4
1501.9714
1482.9745
1237.8383
1517.9417
1503.9840
1519.9336
1239.8517
1523.9650
1167.7384
1253.5887
1355.9954
1351.8574
1553.0115
1652.9866
% Intensity
Mass (m/z)
999.0
1199.4
1399.8
1600.2
1800.6
2001.0

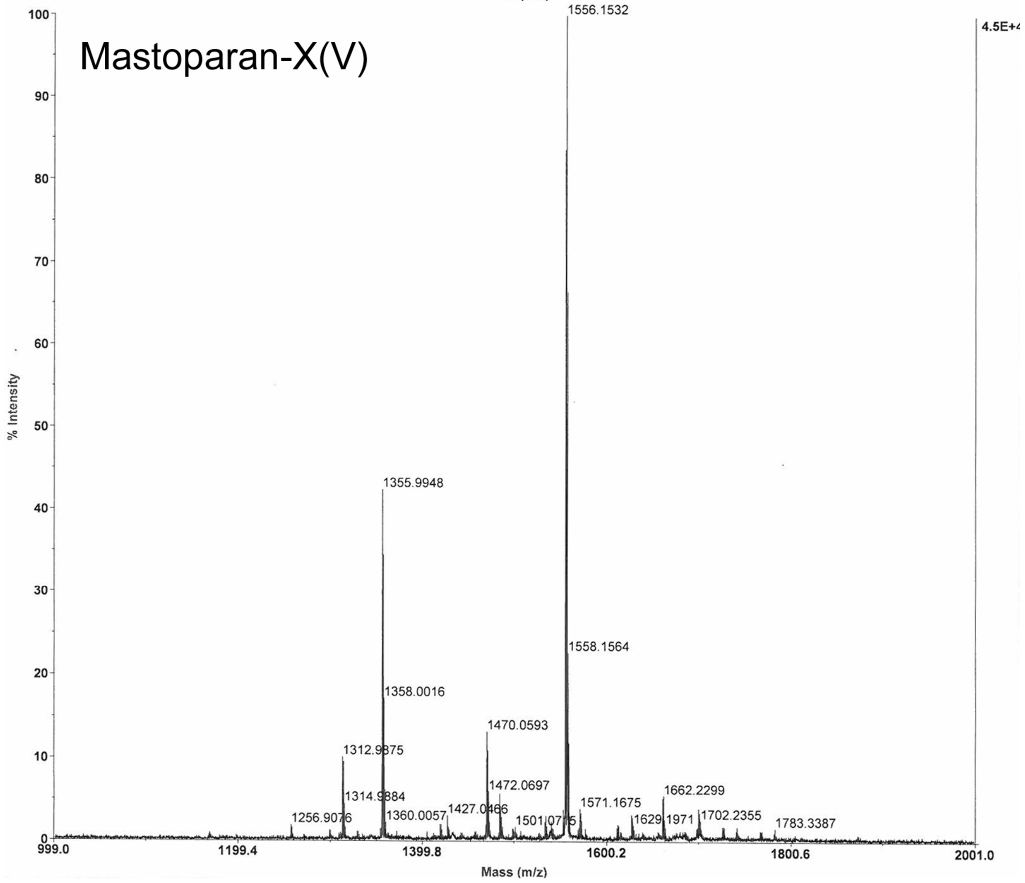
Mastoparan-X(V)
1556.1532
4.5E+4
1355.9948
1558.1564
1358.0016
1470.0593
1312.9875
1472.0697
1662.2299
1314.9884
1571.1675
1256.9076
1360.0057
1702.2355
1783.3387
% Intensity
Mass (m/z)
999.0
1199.4
1399.8
1600.2
1800.6
2001.0

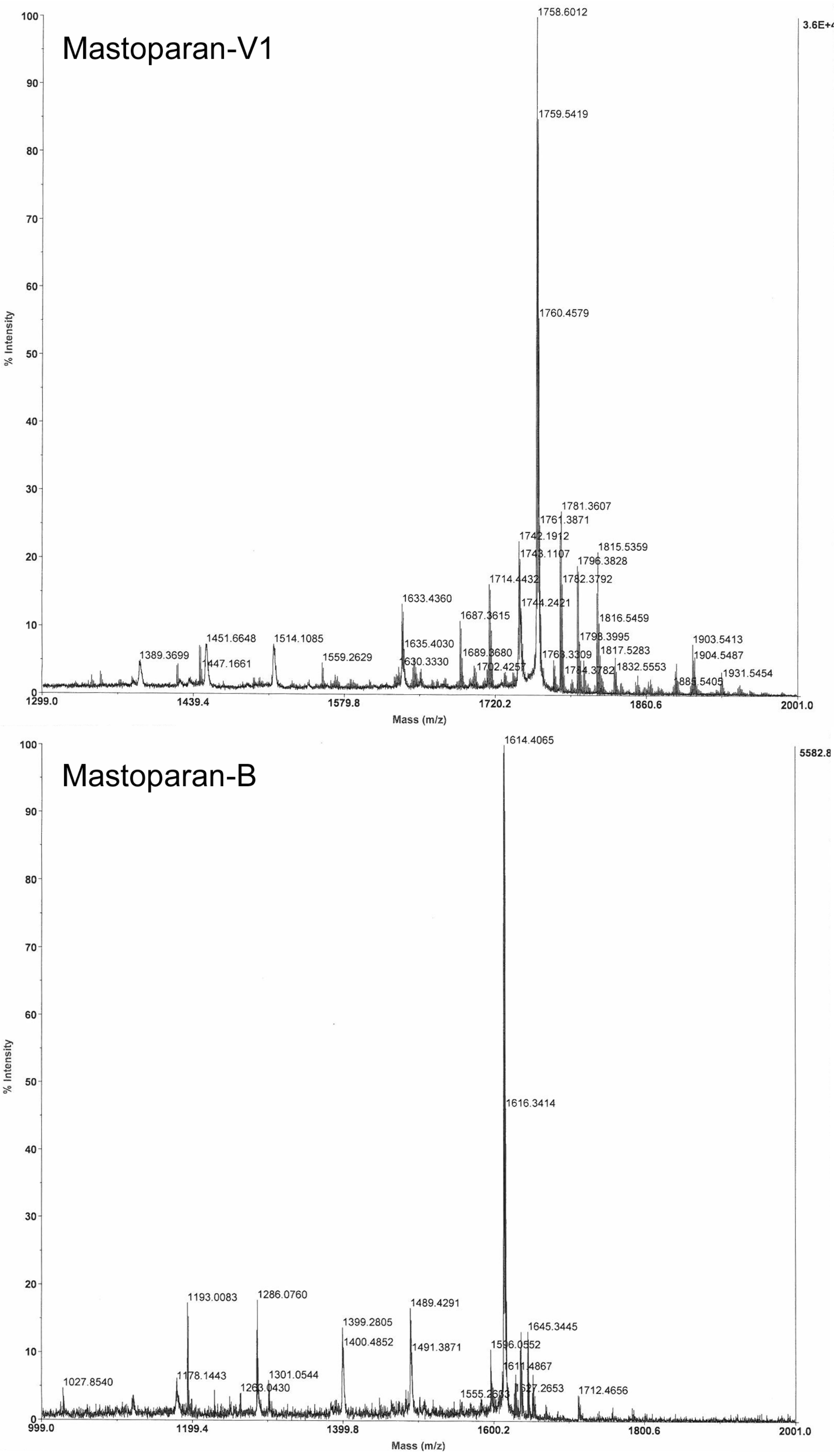

Figure S2. Matrix-assisted laser-desorption ionization-time-of-flight mass (MALDI-TOF MS) analysis of the four mastoparans. The molecular masses of purified peptides were determined using the MALDI-TOF MS spectrometry (KBSI, Ochang, Korea).

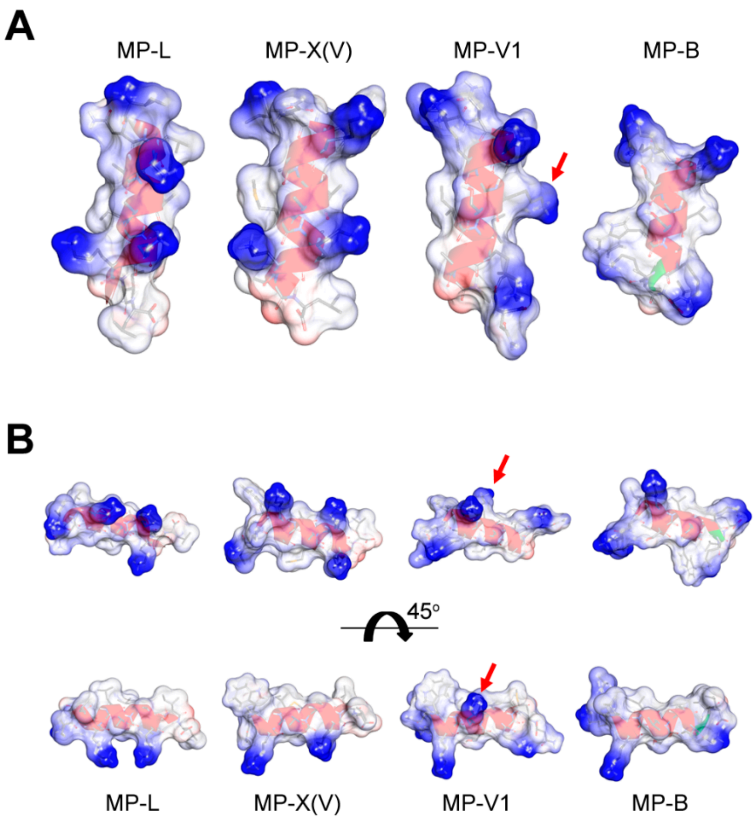

Figure S3. Structural models of mastoparans. The 3D structures of MP-L (PDB ID: 1D7N) and MP-X (PDB ID: 2CZP) were obtained from the RCSB Protein Data Bank (PDB, http://www.rcsb.org/). The structures of MP-V1 and MP-B were predicted by the automated I-TASSER server [1]. The model with the best C-score was selected. The stereochemical quality of the predicted structures was validated using ProSA-web (Protein Structure Analysis) [2]. Energy minimization was performed using GROMACS 5.0.4 package with gromos53a6 force field [3]. Then the structures were immersed in a dodecahedron water box of SPC water molecules to make an aqueous environment. To neutralize the system, counter-ions were added by replacing water molecules. The steepest descent EM algorithm was applied until the maximum force was converged to less than 1000 kJ/mol. During the steps, cut-off value of 1.2 nm was used for short-range electrostatic and van der Waals while long-range electrostatic interactions were calculated using particle mesh Ewald method [4]. The net charges of MP-V1, MP-L, MP-X(V) and MP-B were +4, +3, +3 and +4, respectively. The A and B represent front and side views, respectively, and the blue color in the images indicates a positive charge. The red arrows indicate the 7th lysine in MP-V1.

References

1. Roy, A.; Kucukural, A.; Zhang, Y. I-tasser: A unified platform for automated protein structure and function prediction. *Nat. Protoc.* **2010**, *5*, 725–738.
2. Wiederstein, M.; Sippl, M.J. Prosa-web: Interactive web service for the recognition of errors in three-dimensional structures of proteins. *Nucleic. Acids Res.* **2007**, *35*, W407–W410.
3. Oostenbrink, C.; Villa, A.; Mark, A.E.; Van Gunsteren, W.F. A biomolecular force field based on the free enthalpy of hydration and solvation: The gromos force-field parameter sets 53a5 and 53a6. *J. Comput. Chem.* **2004**, *25*, 1656–1676.
4. Darden, T.; York, D.; Pedersen, L. Particle mesh ewald: An n log (n) method for ewald sums in large systems. *J. Chem. Phys.* **1993**, *98*, 10089–10092.
